# The efficacy of phase I cardiac rehabilitation training based on augmented reality on the self-efficacy of patients undergoing coronary artery bypass graft surgery: A randomized clinical trial

**DOI:** 10.1186/s13102-023-00770-9

**Published:** 2023-11-17

**Authors:** Neda Ghlichi Moghaddam, Mohammad Namazinia, Fatemeh Hajiabadi, Seyyed Reza Mazlum

**Affiliations:** 1 Department of Medical – Surgical Nursing, School of Nursing and Midwifery (MSC Student), Mashhad University of Medical Sciences, Mashhad, Iran; 2 Department of Nursing, School of Nursing and Midwifery, Torbat Heydariyeh University of Medical Sciences, Torbat Heydariyeh, Iran; 3 Department of Medical – Surgical Nursing, School of Nursing and Midwifery, Mashhad University Medical of Medical Sciences, Mashhad, Iran; 4 Nursing and Midwifery Care Research Center, Mashhad University Medical of Medical Sciences, Mashhad, Iran

**Keywords:** Coronary artery disease, Phase I cardiac rehabilitation training, Augmented reality, Self-efficacy

## Abstract

**Background:**

Open-heart surgery is considered one of the primary treatments for severe coronary artery stenosis, but it comes with its own set of complications. However, these complications can be reduced through the implementation of proper cardiac rehabilitation during phase I. This study aimed to examine the impact of phase I cardiac rehabilitation training, using augmented reality, on the self-efficacy of cardiac management in patients undergoing coronary artery bypass grafting.

**Methodology:**

This randomized clinical trial study involved 60 patients who were admitted to the Cardiac Surgery Intensive Care Unit at Ghaem Hospital in Mashhad. The software used in this study consisted of various videos and educational images demonstrating physical exercises for cardiac rehabilitation. The software was developed to train the patients in the intervention group on the rehabilitation program, starting from their admission to the Intensive Care Unit until their discharge from the hospital. The collected data were analyzed using statistical tests such as independent t-test, Mann-Whitney test, paired t-test, chi-square test, as well as descriptive indicators. Cohen’s d was also used to evaluate the magnitude of the effect size.

**Results:**

The findings of this study revealed that the total mean score for cardiovascular management self-efficacy significantly increased during the transfer to the Intensive Care Unit and at the time of discharge. Notably, the increase observed in the intervention group was significantly greater than that of the control group (*P* < 0.001).

**Conclusion:**

The results of this study indicated that implementing early rehabilitation programs, using innovative educational technology like augmented reality, enhanced the self-efficacy of patients undergoing coronary artery bypass grafting. These findings suggest that such programs can be effectively employed as educational tools throughout different stages of cardiac rehabilitation.

**Trial Registration:**

This study was registered in the Iranian Registry of Clinical Trials (no. IRCT20200203046361N1) on 16/02/2020.

## Introduction

The prevalence of cardiovascular diseases has grown globally in recent years [1]. Each year, around 3.6 million men and 3.4 million women worldwide pass away due to cardiovascular disease [2]. In developing countries, this illness is the leading cause of death [3], and in Iran, it is the leading cause of death for both men and women [4–6].

Coronary artery disease is responsible for about 30% of all cardiovascular diseases [7, 8]. One of the effects of this disease is ischemic coronary artery [9]. Angiograms are used to confirm the diagnosis, and treatment options include angioplasty or coronary artery bypass graft surgery (CABG) [10]. Annually, over a million coronary artery graft surgeries are carried out worldwide [11], with the majority of open-heart surgeries in Iran being CABG procedures [12].

In the initial days after undergoing CABG, patients are typically hospitalized in the cardiac surgery intensive care unit [13]. For individuals with ischemic coronary artery, this surgery represents a significant milestone in their lives. However, following the procedure, these patients often encounter various physical and psychological challenges, such as restlessness, insomnia, and limitations on physical activity [14].

Research suggests that the care provided during this critical period can enhance self-efficacy and empower patients to take charge of their own well-being [15, 16]. In the context of illnesses, self-efficacy beliefs have been shown to predict health-promoting behaviors [17–20]. Specifically, in the case of heart diseases, higher levels of self-efficacy are associated with improved physical and mental performance, adherence to treatment, and greater engagement in self-care practices [21, 22].

The choice of training method significantly influences patients’ learning and their willingness to change health-related behaviors, ultimately affecting their self-efficacy [23]. Patients who require additional training in various situations should receive ongoing support.

Cardiac rehabilitation (CR) is recognized as one of the most crucial approaches for enhancing self-efficacy [24, 25]. CR is a structured program designed to improve the health and well-being of individuals who have undergone cardiac procedures, such as CABG surgery. This comprehensive rehabilitation program involves a multidisciplinary team of healthcare professionals, including cardiologists, nurses, exercise physiologists, and dietitians, who collaborate to develop personalized plans for each patient [26, 27]. CR consists of three phases [28]. Research indicates that when phase I CR is implemented effectively, patients experience improved well-being following cardiac surgery and demonstrate enhanced quality of life and self-efficacy after being discharged from the hospital [29].

Augmented reality technology is a relatively new addition to medical education and patient rehabilitation skills training. It combines text, images, and videos with real-world environments [30, 31]. By incorporating physical movements within augmented reality, viewers are encouraged to focus on their training while minimizing distractions from the outside world [18].

Numerous studies have explored the effects of cardiac rehabilitation (CR) on patients who have undergone CABG surgery [32, 33]. A systematic review conducted by de Araújo Pio (2017) examined the impact of CR on postoperative mortality, cardiovascular events, exercise capacity, and quality of life in patients with coronary heart disease, including those who underwent CABG surgery [34]. Another review by Lourens et al. (2022) evaluated the influence of CR on health-related quality of life in patients following CABG surgery. The review revealed that CR interventions were associated with significant improvements in physical function, emotional well-being, and overall quality of life among this population. Furthermore, it emphasized the importance of long-term participation in these programs to sustain positive outcomes [35].

These findings underscore the significance of integrating CR as an essential component of postoperative care for CABG patients, as it enhances their sense of self-efficacy during the recovery process [27, 36, 37].

Surprisingly, there is currently no research available on CR training based on augmented reality, despite the importance of phase I CR and its impact on self-efficacy. Therefore, this study aimed to investigate the effect of phase one CR training using augmented reality on the self-efficacy of patients after cardiac surgery.

## Methods

### Trial Design

**Fig. 1:**
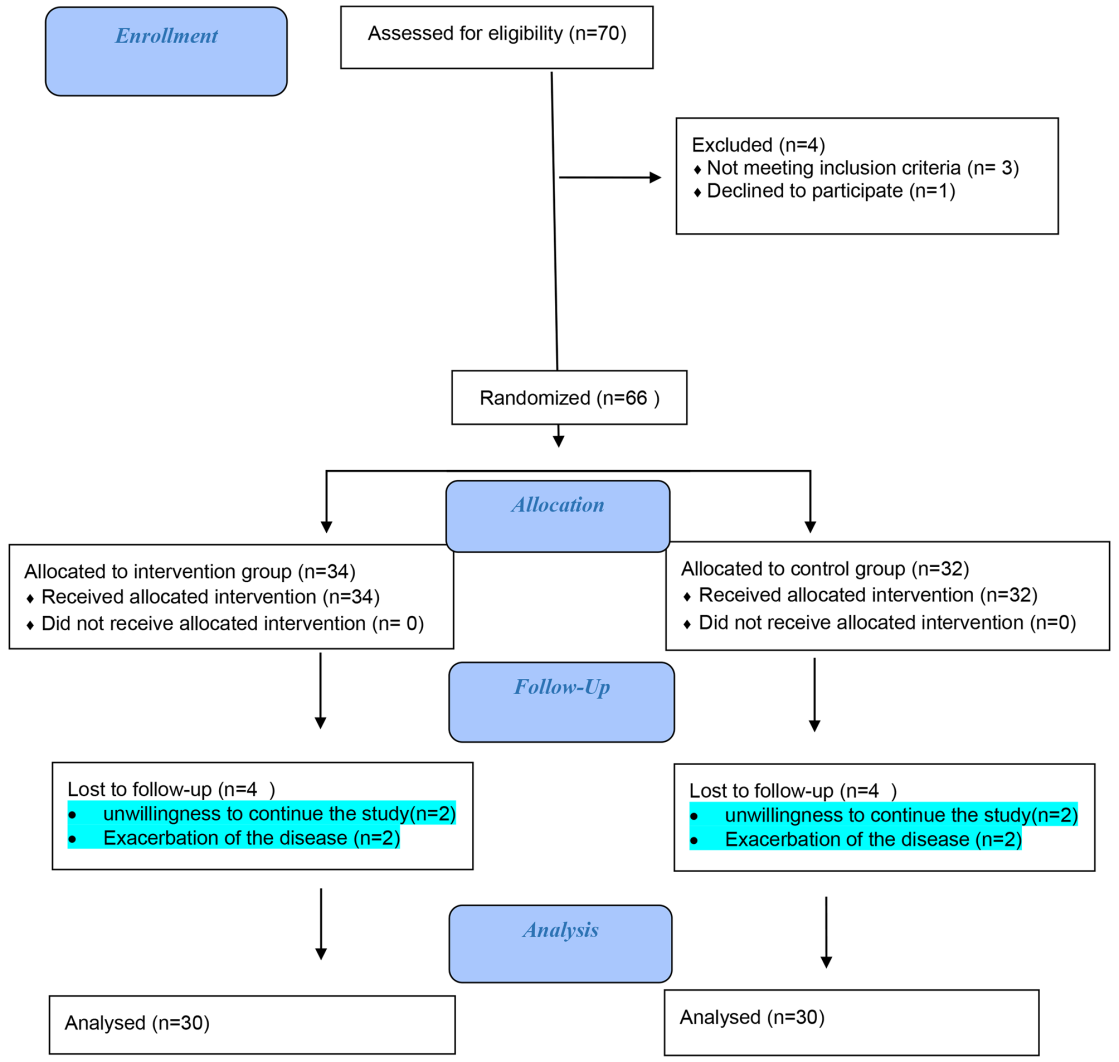
CONSORT Flow Chart of participants

This controlled clinical trial study involved 60 patients admitted to the Ghaem Hospital of Mashhad, Iran. These patients were specifically from the cardiac surgery intensive care unit and were admitted between May 2020 and January 2021 (Fig. 1).

### Participants

The study included patients who met specific inclusion criteria. These criteria required the patients to be between the ages of 18 and 60 and willing to undergo non-emergency coronary artery graft surgery. On the other hand, exclusion criteria included patients who experienced loss of consciousness until the day following surgery, those who did not have a smartphone, individuals with severe postoperative arrhythmias and hemodynamic disorders, and patients who were prohibited by their doctor from participating in rehabilitation.

### Intervention

#### Software production

Prior to the software’s design, extensive research was conducted to prepare its content. This involved reviewing various texts, including articles, reference books, and gathering insights from experienced nurses in specialized care units. The content was then submitted to a panel of 10 specialists for validation, and their suggested revisions were incorporated.

The educational content of the software covered a range of topics, including respiratory diaphragmatic exercises, instructions on physical exercises and their proper execution, discussions and interactions with patients, and encouragement for patients to engage in routine activities. These concepts were primarily presented through instructional videos and engaging animations.

Once the content was finalized, it was handed over to the software development and information technology team for the creation of the software. After the initial software was developed, a specialized validation process was conducted by 10 IT experts to ensure its functionality and effectiveness.

To validate the software, both white-box and black-box testing methods were employed. In black-box testing, users with no knowledge of the software’s internal structure input their desired items and verify the recorded information. The aim is to ensure accurate data recording. White-box testing, on the other hand, requires users to have knowledge of the software’s internal structure and is typically executed by designers or experts. For instance, to assess the software’s speed, various items were selected at different speeds, and the accuracy of the selections was examined.

The next phase involved compatibility testing and security testing. Compatibility testing involved installing the application on multiple Android-based smartphones and tablets to assess its performance on each device. In security testing, a double confirmation method was implemented to ensure accurate recording of each patient’s issues. This involved the patient confirming their selected item by clicking again, reducing the possibility of accidental data entry errors.

The augmented reality software was registered and approved within the electronic services system of the Information Technology Organization of Iran.

To evaluate patient satisfaction with the augmented reality software, the Mobile Application Rating Scale (MARS) was employed.

This scale evaluates the application’s quality and performance across four dimensions: attractiveness (5 questions), functionality (4 questions), aesthetics (3 questions), information (7 questions), and subjective quality (4 questions). Each item in the scale was rated on a 5-point scale. The maximum achievable score was 115, while the minimum acceptable score was set at 23. For a detailed presentation of the results, please refer to (Table 1).

**Table 1:** Mean and standard deviation of dimensions of MARS questionnaire

Variable		Number of questions	Points earned		Maximum score
			Mean ± SD	(%)	
MARS questionnaire dimensions	Attractiveness	5	23.1 ± 1.1	92.4	25
	Function	4	18.2 ± 1.1	91	20
	Aesthetics	3	13.7 ± 0.8	91.3	15
	Information	7	30.2 ± 1.4	86.2	35
	Subjective quality	4	15.1 ± 4.1	75.5	20
The total score of the MARS questionnaire		23	100.4 ± 3.0	87.3	115

#### Phase I cardiac rehabilitation training based on augmented reality

After establishing the necessary agreements with officials at Ghaem Hospital in Mashhad, Iran, the first author of the study initiated the sampling process. In the intervention group, the rehabilitation program training started upon the patient’s entry into the cardiac surgery intensive care unit and continued until their discharge from the unit.

During multiple sessions, augmented reality software was utilized to train patients in physical activities, such as walking around the inpatient ward and climbing stairs. These exercises were done under the direct supervision of the researcher and were individually taught to each patient using the augmented reality software. The duration of physical activity varied based on the patient’s condition and length of hospital stay, ranging from 5 to 10 min. Throughout the rehabilitation sessions, ECG and the perceived exercise intensity were closely monitored and controlled.

In the control group, the rehabilitation training program was implemented using a routine method based on the Ministry of Health protocol. The researcher provided face-to-face training within the unit. Both the intervention and control groups completed the cardiac self-efficacy questionnaire upon admission and at the time of discharge in the special care unit of cardiac surgery.

### Outcomes

In the data collection process, two demographic information questionnaires and a cardiac self-efficacy questionnaire were utilized.

The cardiac self-efficacy questionnaire used in this study was the Cardiovascular Management Self-Efficacy Questionnaire, which was developed by Estka from Italy in 2015. This questionnaire consists of 9 questions, each rated on a 5-point Likert scale, ranging from “completely confident” to “not at all confident.” The questionnaire is composed of three subscales.

The first four questions assess a person’s belief in their ability to quit smoking, maintain proper nutrition, engage in exercise, and avoid stressful situations. This subscale is referred to as self-efficacy of cardiac risk factors. Questions 5 and 6 pertain to a person’s confidence in remembering to take medications correctly, representing self-efficacy of treatment adherence. Lastly, questions 7–9 evaluate a person’s belief in their ability to identify symptoms and signs of disease exacerbation, indicating self-efficacy in symptom recognition.

Each response is assigned a score, with “not confident at all” receiving a score of one, “slightly confident” receiving a score of two, “somewhat confident” receiving a score of three, “fairly confident” receiving a score of four, and “completely confident” receiving a score of five. The total scores range from 9 to 45, with higher scores indicating greater self-efficacy in cardiovascular management [21]. Borzou et al. (2017) evaluated the validity and reliability of this tool in Iran [33]. The patients completed the Cardiovascular Management Self-Efficacy Questionnaire both before and after the intervention.

### Sample size and randomization

The study involved the continuous and purposeful selection of patients who were then randomly assigned to one of two groups. After confirming that they met the inclusion criteria, eligible individuals were divided into intervention and control groups using a random sequence generated by SPSS software. This sequence was kept in a sealed envelope to maintain confidentiality. While it was challenging to blind the participants in this trial, the outcome assessors and statisticians were unaware of the type of intervention, ensuring a level of objectivity.

Since no similar study was found that examined the efficacy of phase I cardiac rehabilitation training based on augmented reality on the self-efficacy of patients undergoing coronary artery bypass graft surgery, a sample size of 10 participants was determined for each group. The sample size was calculated using the mean comparison formula, with a confidence interval of 95% and a test power of 80% for each group, resulting in a total of 20 participants. To account for potential dropout probability, an additional 30 participants were added to each group, representing a 10% increase from the calculated values in the formula.

$$N = {\text{ }}\left( {Z1 - \alpha /2{\text{ }} + {\text{ }}Z1 - \beta } \right)2{\text{ }}\left( {S12{\text{ }} + {\text{ }}S22} \right)/\left( {X1 - X2} \right)2$$

$${Z_{1 - \alpha /2}} = {\text{ }}196$$

$${Z_{1 - \beta }} = {\text{ }}0.85$$

$${X_1} = 7$$

$${S_1} = 1.5$$

$${X_2} = {\text{ }}8.3$$

$${S_2} = 1.8$$

### Statistical methods

After data collection and sampling, the collected data were analyzed using SPSS 21. Various statistical tests were employed, including independent t-test, Mann-Whitney test, paired t-test, and chi-square test. These tests were conducted with a 95% confidence level to ensure statistical significance. Descriptive indicators such as mean, standard deviation, and frequency were also used to provide a comprehensive overview of the data. Cohen’s d was also used to evaluate the magnitude of the effect size, calculated by standardized mean difference, with g > 0.2 to 0.5 = small effect size, g > 0.5 to 0.8 = medium effect size and g > 0.8 = large effect size [38].

## Results

In the study, male patients accounted for 22 cases (73.3%) in both the intervention and control groups. The mean age in the intervention group was 58.2 ± 8.3 years, while in the control group it was 59.1 ± 11.7 years (Table 2).

**Table 2:** Frequency distribution of demographic variables of patients undergoing coronary artery graft surgery in two test and control groups

Variable		Group		The test result
		Intervention	Control	
		*N* = 30	*N* = 30	
Age (Years) (Mean ± SD)		58.2 ± 8.3	59.1 ± 11.7	**P = 0.733
Gender (%)*N*	Male	22(73.3)	22(73.3)	*P = 0.001
	Female	8(26.7)	8(26.7)	
Marital Status (%)*N*	Single	1(3.3)	1(3.3)	***P = 0.768
	Married	29(96.7)	29(96.7)	
Smoking (%)*N*	No	23(76.7)	24(80.0)	*P < 0.001
	Yes	7(23.3)	6(20.0)	
Education level (%)*N*	Elementary	9(30.0)	16(53.3)	*P = 0.235
	Cycle	16(53.3)	9(30.0)	
	Diploma	5(16.7)	4(13.3)	
	Academic	0.00	1(3.3)	
Height, weight and body mass index	Height (cm)			**P = 0.908
		170.8 ± 9.9	173.1 ± 10.8	
	Weight (kg)	75.1 ± 11.7	76.7 ± 12.2	
	Body mass index (kg/m2)	25.7 ± 3.1	25.8 ± 5.1	
History of hospitalization in CCU (%)*N*	No	22(73.3)	26(86.7)	*P = 0.333
	Yes	8(26.7)	4(13.3)	

* Chi-square ** independent t- test *** Exact chi-square

Regarding the length of stay in the ICU for patients who underwent coronary artery surgery, the analysis of the data showed that it was 1.3 ± 0.5 days in the intervention group and 1.4 ± 0.5 days in the control group (*P* = 0.251) (Table 3).

**Table 3:** Frequency distribution of data related to coronary artery graft surgery of research units in two test and control groups

Variable		Group		Result
		Intervention	Control	
		*N* = 30	*N* = 30	
Systole blood pressure(mmHg) (Mean ± SD)		132.7 ± 20.5	137.2 ± 17.3	***P = 0.443
Diastole blood pressure(mmHg) (Mean ± SD)		82.2 ± 9.1	77.5 ± 11.7	****P = 0.089
Type of surgery	On pomp	16(83.3)	18(60.0)	*P = 0.794
	Off pomp	14(46.7)	12(40.0)	
Underlying conditions disease *N*(%)	No	10(33.3)	36.7))11	*P < 0.001
	Yes	20(66.7)	19(63.3)	
Past medical history *N*(%)	Past medical history	4(20.0)	8(42.1)	**P = 0.319
	Past medical history			
	Hyperlipidemia	4(20.0)	2(10.5)	
	HTN	12(60.0)	8(42.1)	
	Musculoskeletal Disorder	0(0.0)	1(5.3)	
Duration of surgery (hours) (Mean ± SD)		3.7 ± 0.4	3.8 ± 0.3	***P = 0.464
Length of stay in ICU (days) (Mean ± SD)		1.3 ± 0.5	1.4 ± 0.5	***P = 0.251
Ejection fraction (%) (Mean ± SD)		46.5 ± 6.8	41.5 ± 13.1	***P = 0.276

* Chi-square **Exact chi-square *** Mann–Whitney U test **** independent t- test

The results of the current study indicated a significant improvement in the total mean (SD) score of cardiovascular management self-efficacy in the intervention group. This improvement was observed at the time of transfer to the ICU and at the time of discharge, following the implementation of the phase I cardiac rehabilitation program based on augmented reality. An independent t-test confirmed the statistical significance of this difference (*P* < 0.05) (Table 4).

**Table 4:** Comparing the self-efficacy and its dimensions in the studied patients before and after the intervention, separating the two groups

Variable			Intervention	Control	Effect Size	*P*
			*N* = 30	*N* = 30		
Cardiovascular management self-efficacy total score (Mean ± SD)		Admission	26.3 ± 5.5	23.0 ± 3.9	0.69	***0.034
		Transfer to ICU	28.4 ± 5.0	24.9 ± 2.1	0.91	****0/001
		Discharge	29.9 ± 8.8	26.8 ± 3.1	0.47	***0.020
Dimensions of self-efficacy	Self-efficacy of cardiac risk factors (Mean ± SD)	Admission	12.6 ± 2.7	10.3 ± 1.8	1.02	***0.001
		Transfer to ICU	14.9 ± 1.9	11.2 ± 2.0	1.87	***P < 0.001
		Discharge	14.1 ± 3.2	11.9 ± 2.0	0.80	***P < 0.001
	Treatment adherence self-efficacy (Mean ± SD)	Admission	5.5 ± 1.7	5.1 ± 1.4	0.25	***0.445
		Transfer to ICU	6.2 ± 1.9	5.5 ± 1.2	0.43	***0.112
		Discharge	7.0 ± 2.3	6.0 ± 1.4	0.52	****0.049
	Self-efficacy in symptom recognition (Mean ± SD)	Admission	8.2 ± 1.7	7.6 ± 2.1	0.29	***0.160
		Transfer to ICU	7.4 ± 2.7	8.2 ± 2.0	-0.33	****0.197
		Discharge	8.9 ± 4.1	8.9 ± 1.8	-0.01	***0.470

*** Mann–Whitney U test **** independent t- test

## Discussion

The results of the present study indicated that the intervention group had a higher total mean score of cardiovascular management self-efficacy compared to the control group at the time of transfer to the ICU. This difference was observed during phase I cardiac rehabilitation (CR) training, which utilized augmented reality. Furthermore, the intervention group showed a significant increase in discharge rates compared to the control group.

This finding is consistent with a study conducted by Mohebbi et al. (2018) that compared the effect of the CR training program using two multimedia and face-to-face methods on self-efficacy and spirometry indicators in patients undergoing coronary artery graft surgery. The study demonstrated that both multimedia and face-to-face training methods were effective in improving self-efficacy, with the multimedia method having a greater impact [39]. The findings of this study were in agreement with those of the present one.

One contributing factor to this consistency is the use of educational videos during phase I CR training. Augmented reality technology, as demonstrated in the current study, offers an engaging multimedia approach that can be accessed in natural settings and tailored to individual patient needs. This expands the possibilities of traditional rehabilitation methods when combined with audio or text-based training.

The findings of the study conducted by Borzou et al. (2018) were consistent with the results of our current study. Their study examined the effects of the first phase of CR training on self-efficacy among patients undergoing coronary artery bypass graft surgery. The study demonstrated that self-efficacy scores in all aspects were significantly different between the intervention group and the control group at discharge and one month after discharge [40]. One of the contributing factors to this agreement is the phase I CR training, which consisted of theoretical and practical sessions. However, this method followed a traditional rehabilitation approach, which could be slower and less effective compared to CR exercises done by patients who possessed self-care knowledge and higher motivation. In contrast, the augmented reality system allows patients to carry out the rehabilitation program with greater focus and accuracy. It also helps to control distractions that may be present in actual medical environments [41].

Furthermore, Wang et al. (2016) examined the effects of multimedia training on exercise regimens, heart rate improvement, and self-efficacy in walking. The findings of their study align with our current study, as they demonstrated that the multimedia training program resulted in increased heart rates in the multimedia group compared to the control group. Additionally, similar to our findings, an increase in self-efficacy (*P* = 0.002) was observed in the intervention group, and this improvement persisted for one month (*P* = 0.001). However, the improvement in heart rate was only observed until the time of discharge [32]. One of the reasons for these outcomes was the use of multimedia instruction specifically designed to teach sports activities. It is important to note that the self-efficacy assessed in Wang et al.’s study focused on activity-based self-efficacy, specifically walking self-efficacy. In our current study, augmented reality technology was employed for training purposes, and the patients responded very positively to it. This change in response may be attributed to the attractiveness, motivation, and therapeutic value that augmented reality technology brings to the rehabilitation process [42]. Additionally, the use of augmented reality systems has been shown to accelerate recovery, reduce costs, and have a significant impact on rehabilitation outcomes [29].

The study conducted by Sanayi et al. (2014) found that a family-centered empowerment program could improve a patient’s self-efficacy and self-esteem during coronary artery bypass surgery [43]. This aligns with our study, where augmented reality software was used to instruct patients in the first stage of cardiac rehabilitation from admission to discharge.

Warei et al. (2014) found that two sessions of peer-to-peer training significantly increased the self-efficacy of patients undergoing coronary artery bypass graft (CABG) surgery [44]. These findings are consistent with our current study, as both studies involved the same participants who received training during their hospital stay. However, it is important to note that the teaching methods and content differed between Warei et al.’s study and our own. Therefore, a direct comparison of the effectiveness of the educational methods used in the two studies cannot be made. In our study, we assessed the self-efficacy of patients using Sullivan’s CSE questionnaire (1998).

Interakamhang et al. (2013) demonstrated how a comprehensive cardiac rehabilitation (CR) program, incorporating psychological and educational interventions, could improve psychological aspects such as self-efficacy, self-management, self-care, and quality of life in patients undergoing coronary artery graft surgery [45]. These findings align with the results of our own study. In our study, the CR program was implemented early, starting from the patients’ admission to the department, which contributed to the enhancement of general health and self-efficacy in these patients [46]. However, the difference lies in the use of augmented reality software to deliver these programs in our study, and the assessment of psychological support was done using the coping quality questionnaire.

Lian et al. (2020) conducted a study focusing on comprehensive and early rehabilitation programs that included physical exercises starting from the time of patients’ hospital admission. They found that these programs led to improvements in the physical condition and self-efficacy of patients after coronary artery bypass graft (CABG) surgery [47]. These findings are consistent with the results of our present study. The increase in self-awareness and self-assurance among patients, leading to improved physical independence, may contribute to this agreement.

In a related context, a study on the use of augmented reality in wrist rehabilitation for stroke patients demonstrated that the system enhanced hand movement performance in these patients [48].

The present study demonstrated that phase I cardiac rehabilitation training utilizing augmented reality was effective in improving postoperative self-efficacy. Additionally, it was found to be a cost-effective training method for various stages of cardiac rehabilitation. However, it is important to acknowledge the limitations of this study. One limitation was the inability of some patients to do exercises. Additionally, technical issues such as problems with installing or loading the software could have hindered the smooth implementation of the training program. Patients’ dissatisfaction with illegibility or difficulties in perceiving the font or images of the augmented reality software may have also affected their experience. Furthermore, individual differences among the patients, including their cultural background, could have influenced their ability to complete the questionnaires accurately.

## Conclusion

The study findings indicated that using augmented reality in phase I CR training enhanced postoperative self-efficacy by increasing patients’ engagement with the treatment plan and prioritizing the learning process. Moreover, the use of a mobile phone, even without internet connectivity, proved to be a cost-effective training approach that can be beneficial at different stages of CR.

## Data Availability

The datasets generated in the present study are available from the corresponding author upon reasonable request.

## List of Abbreviations

CR: Cardiac rehabilitation
CABG: Coronary artery bypass graft surgery
